# The RapidIO Routing Strategy Based on the Double-Antibody Group Multi-Objective Artificial Immunity Algorithm

**DOI:** 10.3390/s22030914

**Published:** 2022-01-25

**Authors:** Yanming Fu, Youquan Jia, Baohua Huang, Xing Zhou, Xiaoqiong Qin

**Affiliations:** School of Computer, Electronics and Information, Guangxi University, No. 100 University East Road, Nanning 530004, China; fym2005@126.com (Y.F.); bhhuang66@gxu.edu.cn (B.H.); chowhin1@icloud.com (X.Z.); xqiongqin@126.com (X.Q.)

**Keywords:** double-antibody groups, adaptive crossover, adaptive mutation, routing strategy

## Abstract

The RapidIO standard is a packet-switching interconnection technology similar to the Internet Protocol (IP) conceptually. It realizes the high-speed transmission of RapidIO packets at the transport layer, but this greatly increases the probability of network blocking. Therefore, it is of great significance to optimize the RapidIO routing strategy. For this problem, this paper proposes a Double-Antibody Group Multi-Objective Artificial Immune Algorithm (DAG-MOAIA), which improves the local search and global search ability of the population by adaptive crossover and adaptive mutation of the double-antibody groups, and uses co-competition of multi-antibody groups to increase the diversity of population. Through DAG-MOAIA, an optimal transmission path from the source node to multiple destination nodes can be selected to solve the Quality Of Service (QoS) problem during data transmission and ensure the QoS of the RapidIO network. Simulation results show that DAG-MOAIA could obtain high-quality solutions to select better routing transmission paths, and exhibit better comprehensive performance in all simulated test networks, which plays a certain role in solving the problem of the RapidIO routing strategy.

## 1. Introduction

The advent of the 5G era and the concept of the ’Internet of Everything’ have profoundly accelerated the layout of the Internet of Things (IoT) [1]. Both wireless sensor-based intelligent interconnection technology and the embedded systems-based interconnection technology have been developed rapidly [2,3]. RapidIO, as a kind of embedded high-performance I/O interconnection technology, can be used for signal processing and data transmission, in which data transmission is mainly implemented at the transport layer [4,5]. The underlying structure of RapidIO is similar to the TCP/IP four-layer model [6], including: logical layer, transport layer, and physical layer. Similarly, high-speed transmission of data packets is realized at the transport layer similar to the TCP/IP four-layer model. Moreover, while high-speed data transmission is carried out at the transport layer, the RapidIO network will also meet the problem of packet loss caused by excessive traffic and network congestion [7]. Therefore, it is especially necessary to choose the appropriate RapidIO routing strategy to guarantee the network to transport data quickly and safely.

In traditional networks, only the best-effort service is provided for data transmission without considering the comprehensive performance of transmission cost, packet loss, delay, etc., which will increase the chances of network congestion to a certain extent, and even lead to the problem of network collapse [8]. To avoid or alleviate congestion problems when transferring large amounts of data at high speeds over RapidIO networks, the RapidIO network has higher requirements on QoS such as transmission cost, packet loss, delay etc. [9]. QoS is a technology used to meet network requirements such as transmission cost, packet loss, and delay during packet transmission. The QoS mechanism can ensure the selection of better paths for packets during transmission, allowing the network to operate efficiently [10,11]. Therefore, the problem of the RapidIO routing strategy is actually a QoS multi-constraint routing problem (QoS-MCRP), whose main objective is to find a multicast tree satisfying the QoS constraints [12], where the multicast tree covers the source node and all the destination nodes, so that the packets spend the least cost during the network transmission. This is also equivalent to finding a minimal Steiner Tree with QoS constraints [13]. It has been proved that solving the problem of minimal Steiner tree is actually a Non-deterministic Polynomial Complete (NPC) problem [14], and the most commonly used algorithm for solving it is the meta-heuristic intelligent algorithm.

In recent years, a series of meta-heuristic intelligent algorithms have been used to solve QoS-MCRP. Ying Xu et al. [15] proposed the multi-objective jumping particle swarm algorithm (MOJPSO) for QoS-MCRP. The MOJPSO performs four jumps, i.e., the inertial, cognitive, social and global jumps, to guide particles to move to a better position to create a better search space, solving the problems of randomness and the occurrence of stagnation in the traditional PSO algorithm, enabling the algorithm to jump out of the limits of local optimal. Fei Li et al. [16] proposed the Quantum Ant Colony Multi-Objective Routing Algorithm (QAC-MORA) for QoS-MCRP. In the QAC-MORA, to increase the solution space of the algorithm, the quantum bits are introduced to represent node pheromones, and quantum gates are rotated to update the pheromone. In such a way, the QAC-MORA overcomes the disadvantage of traditional Ant Colony Algorithm falling local optimum and can find a better routing path for the QoS-MCRP. Mohammed Mahseur et al. [17] proposed an Improved Quantum Chaotic Animal Migration Optimization Algorithm (IQCAMOA) to solve the QoS-MCRP. The IQCAMOA improves the Animal Migration Optimization Algorithm (AMOA) using quantum representation to the solutions and introducing chaotic map to determine the random numbers, increasing the diversification and intensification of the algorithm and avoiding the problems of AMOA that easily fall into the local optimum. Yassine Meraihi et al. [18] proposed an Improved Chaotic Binary Bat Algorithm (ICBBA) for the QoS-MCRP. The ICBBA enhances the performance and the robustness of the Binary Bat Algorithm (BBA) and ensures the diversity of the solutions by introducing the logistic map and the trend map to determine the parameter of the pulse frequency, and using a dynamic formulation to update the parameter of the loudness. Qing Liu et al. [19] proposed an Encoding-Free Non-Denoted Sorting Genetic Algorithm (EF-NSGA) for QoS-MCRP. The EF-NSGA realizes a coding-free scheme by designing a new gene structure, and putting forward new crossover and mutation operators to increase the performance of the algorithm. The EF-NSGA can quickly obtain a high-quality Pareto front and overcome the slow convergence speed of the traditional Genetic Algorithms. Huanlai Xing et al. [20] proposed a Multi-Objective Artificial Bee Colony Algorithm (MOABCA). The MOABCA integrates two schemes: the elitism-based bee food source generation scheme for scout bees and the Pareto local search operator scheme. Through these two schemes, the local search ability and global search ability of the algorithm is improved, and the diversity of the population is increased at the same time, solving the problem of falling into the local optimum of traditional Bee Colony Algorithm (BCA).

The above research shows that the QoS-MCRP is a discrete problem and relatively complex. In the process of solving QoS-MCRP, the traditional meta-heuristic algorithm cannot be used directly; the encoding method needs to be modified. In addition, the traditional meta-heuristic algorithm easily falls into the local optimum and difficult to obtain a better solution. In response to this issue, this paper makes the following contributions. This paper proposes DAG-MOAIA, which adopts a new encoding method, two-cell coding [21], and designs adaptive crossover operators and adaptive mutation operators in the multi-objective problem for the first time. Then, for the RapidIO routing strategy problem existing in the RapidIO network, a QoS-MCRP model is established, and the proposed DAG-MOAIA is adopted to deal with the problem model. The main idea of DAG-MOAIA is that during the immunization of antibody population, the probability of crossover between antibodies and the probability of mutation of antibodies are determined by a variety of factors, which enable the probability of crossover and mutation to adaptively change under different conditions. Where antibody Group 1 focuses on local search, it needs to increase the crossover probability between similar antibodies to jump out of the local optimum by adaptive crossover operator; antibody Group 2 needs to increase the solution space to focus on a global search by means of an adaptive mutation operator. Finally, a new generated antibody group is introduced to achieve the co-competition of multiple groups. By the concept of survival of the fittest, the outstanding antibodies will be passed to the next generation, which increases the superiority of the population based on ensuring the diversity of the population. The experimental results show that the DAG-MOAIA can find the optimal solution and guarantee the high quality of the solution. Therefore, the DAG-MOAIA can well solve the QoS multi-constraint routing problem and be applied to the RapidIO routing strategy.

The remainder of this article is organized as follows. Section 2 introduces the problem background of the RapidIO network, and builds related problem models. Section 3 demonstrates the DAG-MOAIA proposed in this paper. Section 4 exhibits a tree-shaped antibody and applies the operations of DAG-MOAIA to the proposed problem model. Section 5 analyzes the data results of the simulation experiment. Section 6 has a discussion about the work.

## 2. Problem Formulated

### 2.1. Problem Description

**Figure 1:**
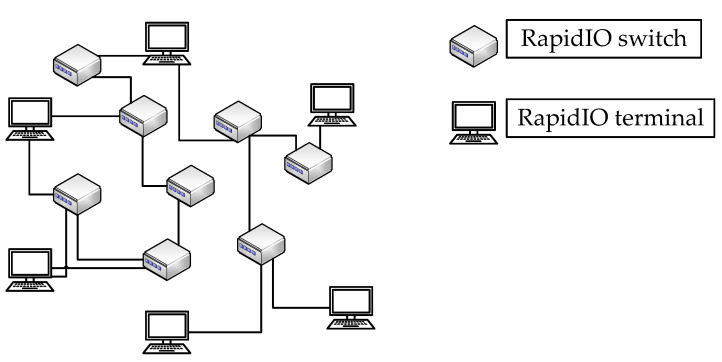
Topology of the RapidIO network.

As is shown in Figure 1, the RapidIO network is mainly composed of switches and terminal devices [22]. Its topology is very flexible. In addition to connecting with switches, the terminals can also be connected to each other. Throughout the RapidIO network, the RapidIO network is used to control and implement the end-to-end transmission of data packets, where the packets mainly contain the information of source ID and destination ID. The switches play the role of forwarding. Each port of the switch has a built-in routing table. According to the destination ID provided, packets are routed from the input port to different output ports by means of routing table mapping. The terminals are matched with the unique device identifier ID as the source or destination of the packet. Apart from that, the terminals play the role of encapsulating and parsing the packet. The workflow of the entire RapidIO network is that the terminal initiates a transmission request, and the switch realizes forwarding to realize data transmission between the terminals.

In the RapidIO network, delay, packet loss, and transmission cost are the most common QoS problems [23]. The aim of QoS-MCRP is to find a multicast tree that satisfies all these constraints, so that data packets can be transmitted end to end on the multicast tree with the least cost.

### 2.2. Problem Modeling

The communication between the terminals in RapidIO is full duplex communication [24]. In the topology of RapidIO network, the structure in the network can be represented by an undirected assignment graph G=<V,E>, where $\left.V=\left\{v_{i}\;\right|\;i=1,2,3,\ldots ,n\right\}$, representing the set of nodes in the network, *n* denotes the number of nodes in the network. $\left.E=\left\{e_{ij}\right|\;e_{ij}=\left(v_{i},v_{j}\right);\;v_{i},v_{j}\in V\right\}$, which represents all communication links in the network. Each link $e_{ij}$ has three properties: Pack_loss, Delay, Cost. For a given source node s∈V, destination node t∈T, where $T\subseteq \left\{V-\left\{s\right\}\right\}$, find an optimal multicast tree $T\left(s,T\right)$ from source node *s* to destination nodes *T*.

When DAG-MOAIA is applied to the RapidIO routing strategy problem, the antibody itself represents a multicast tree $T\left(s,T\right)$. On the multicast tree, the measurement of the transmission consumption of each multicast tree is related to all the nodes and links of the multicast tree, and the calculation method is mainly to add the cost of the node and the cost of the link. The same is true for the delay in computing the multicast tree. When measuring the packet loss rate on the multicast tree, it is calculated by subtracting the product of the packet loss rate of all nodes on the multicast tree from 1. Therefore, the following relevant functions are defined: Packet loss rate function: $Pack\_loss\left(T\left(s,T\right)\right)$, delay function: $Delay\left(T\left(s,T\right)\right)$, transmission cost function: $Cost\left(T\left(s,T\right)\right)$. The functional relationship is shown in Equation (1).

(1) $$\left\{\begin{array}{c} fc=Cost\left(T\left(s,T\right)\right)=\sum_{e\in T(s,T)}Cost\left(e\right)+\sum_{v\in T(s,T)}Cost\left(v\right)\\ fd=\;Delay\left\{T\left(s,T\right)\right.=\sum_{e\in T(s,T)}Delay\left(e\right)+\sum_{v\in T(s,T)}Delay\left(v\right)\\ fp=\;Pack\_loss\left(T\left(s,T\right)\right)=1-\prod_{e\in T(s,T)}Pack\_loss\left(e\right) \end{array}\right.$$

In the DAG-MOAOA, the antibody is a multicast tree covering the source node and all destination nodes. The fitness function is defined in Equation (2)

(2) $$\;f\left(T\left(s,T\right)\right)=\left(fc,fd,fp\right)$$

The QoS-MCRP model of the RapidIO routing strategy problem can be described as a multi-objective optimization problem shown in Equation (3).

(3) $$minimize\;y=\;f\left(T\left(s,T\right)\right)=\left(fc,fd,fp\right)$$

### 2.3. Problem-Model Analysis

When optimizing a multi-objective problem, the sub-objectives conflict with each other. The optimization of one objective will cause the performance degradation of other objectives [25]. Therefore, while dealing with multi-objective problems, it is necessary to take into account the changes of each objective at the same time. The concept of non-dominated solutions is introduced [26] in the QoS-MCRP model of the RapidIO routing strategy problem mentioned in Equation (3), for the given antibody *u*, *v*, when $f_{u}c<f_{v}c$, $f_{u}d<f_{v}d$, $f_{u}p<f_{v}p$, denoted $f\left(u\right)<\;f\left(v\right)$. In addition, when and only when $f\left(u\right)<\;f\left(v\right)$, we call *u* dominates *v*, denoted u≻v, indicating that there is at least one objective component of u in the minimum optimization problem that is smaller than *v*, and no other objective component is larger than *v*. If and only if *u* does not exist to be dominated by other solutions, then *u* is called the non-dominated solution or Pareto optimal solution. For the given subset $Q=\left\{u_{1},u_{2},u_{3},\ldots ,u_{m}\right\}$, where $u_{m}$ is the Pareto optimal solution, and $u_{1},u_{2},u_{3},\ldots ,u_{m}$ are non-dominated each other, then such a subset is called the Pareto sets. The set of objective value vectors corresponding to each solution in Pareto sets is called the Pareto front, abbreviated as PF. The process of solving a multi-objective optimization problem is the process of finding Pareto sets.

The QoS-MCRP model of the RapidIO routing strategy problem does not take constraint parameters as limiting conditions of a certain range or take multiple optimization objectives as penalty function to form a single objective through weighting [27,28]. This paper optimizes several constraint parameters at the same time, and finally produces a set of non-dominated solutions, i.e., Pareto sets, from which the satisfactory solutions can be selected. Compared with the single objective, which only finds its own optimal solution under certain constraints, the general solution is not the optimal solution for the multiple objectives, and the meaning of optimization is not reflected. In contrast, this paper has the flexibility to optimize all the objectives.

## 3. DAG-MOAIA

The immune system is the defense system of mammals against foreign viruses. Animals may suffer various injuries during their lives, and the immune system will take effect at this time. The artificial immune algorithm (AIA) is inspired by the natural immune system [29]. It uses some characteristics of the biological immune system to solve some combinatorial optimization problems and has strong information-processing capabilities [30].

The diversity of antibody population will be weakened while the AIA evolves to a late stage, which will lead to premature convergence and stagnant search. To address this problem, this paper proposes a DAG-MOAIA. The main idea of DAG-MOAIA is shown as follows. The two antibody groups focus on different directions, where antibody Group 1 focuses on local search, and antibody Group 2 focuses on global search. In addition, then, the co-competition among multi-antibody groups is implemented by introducing the new generated antibody group, thus further increasing the diversity of antibody population. The main operations of DAG-MOAIA include clone selection, adaptive crossover, adaptive mutation, and clone inhibition.

### 3.1. The Operations of DAG-MOAIA

This section describes the idea of implementing clone selection, adaptive crossover, adaptive mutation, clone inhibition of DAG-MOAIA.

#### 3.1.1. Clone Selection

Clone selection is an important operation for the generation of antibody Group 1 and antibody Group 2, the main purpose of which is to find better antibodies from the initial antibody population and put them into the next-generation population. This paper simulates the realization idea of the binary tournament selection [31]. Two antibodies are selected from the initial antibody population and compared. The relatively optimal individual is selected to be added to antibody Group 1 or antibody Group 2. Generally, the size of antibody Group 1 and antibody Group 2 is 1/2 of the initial antibody population.

#### 3.1.2. Adaptive Crossover

Crossover operation is an important operation to obtain new antibodies and avoid falling into the local optimum. This paper designs an adaptive crossover operator. When two antibodies are similar, the crossover probability will be increased. When the difference between two antibodies is great, their crossover probability will be reduced. All individuals of the antibody group are performed by non-dominated sorting [32], and each antibody has the attribute of Pareto rank. The crossover probability of an antibody is determined by the average Pareto rank of the current antibody group and antibody itself. When the Pareto rank of two antibodies is low and there is not much difference, the crossover probability of the two antibodies will be enhanced, which helps to avoid falling into local optimum on the one hand, and ensures that the excellent characteristics of the antibody can be passed on to the next generation on the other hand. When the Pareto rank between antibodies is quite different, and both greater than the average of the current Pareto rank, their crossover probability should be decreased. The crossover method used in this article is the double-body crossover method. We randomly select antibody a and b from the antibody population. According to their Pareto rank, the crossover probabilities of antibody Group 1 and antibody Group 2 are calculated as shown in (4) and (5).

(4) $$P_{c1}=\left\{\begin{array}{c} \frac{P_{cmax1}}{\left.{\left|P\right.}_{a}-P_{b}\right|}\;\;\;\;if\;\frac{P_{a}+P_{b}}{2}\leq P_{avg},P_{a}\neq P_{b}\;\;\;\;\;\;\;\;\;\;\;\;\;\\ P_{cmax1}\;\;\;\;if\;\frac{P_{a}+P_{b}}{2}\leq P_{avg},P_{a}=P_{b}\;\;\;\;\;\;\;\;\;\;\;\;\\ P_{cmin1}\;\;\;\;if\;\frac{P_{a}+P_{b}}{2}>P_{avg}\;\;\;\;\;\;\;\;\;\;\;\;\;\;\;\;\;\;\;\;\;\;\;\;\;\;\; \end{array}\right.$$

(5) $$P_{c2}=\left\{\begin{array}{c} \frac{P_{cmax2}}{\left.{\left|P\right.}_{a}-P_{b}\right|}\;\;\;if\;\;P_{a}\neq P_{b}\\ P_{cmin2}\;\;\;\;\;\;if\;P_{a}=P_{b} \end{array}\right.$$

where $P_{c1}$ denotes the crossover probability of antibody Group 1, $P_{c2}$ denotes the crossover probability of antibody Group 2, $P_{a}$ denotes the Pareto rank of antibody *a*, $P_{b}$ denotes the Pareto rank of antibody *b*, and $P_{avg}$ denotes the average Pareto rank in the current antibody group, $P_{max1}$ denotes the maximum crossover probability of antibody Group 1 and $P_{min1}$ denotes the minimum crossover probability of antibody Group 1, $P_{max2}$ denotes the maximum crossover probability of antibody Group 2 and $P_{min2}$ denotes the minimum crossover probability of antibody Group 2.

#### 3.1.3. Adaptive Mutation

The mutation operation can assist in crossover operation to generate new antibodies and is associated with global search capability. In this paper, adaptive mutation is used in combination with adaptive crossover to enable the algorithm to have both local search capability and global search capability. Antibody Group 1 focuses on local search and the mutation operation uses the ordinary mutation, i.e., the probability of mutation is a fixed value; Antibody Group 2 focuses on global search, then the algorithm needs to create new search hyperplanes that go beyond the local scope, which means that a new mutation method needs to be adopted to increase the mutation probability of antibody population. This paper puts forward an adaptive mutation operator in which the mutation probability of antibody Group 2 is determined by its own dispersion and the dispersion of antibody Group 1, where the dispersion represents the difference between the largest Pareto rank and the smallest Pareto rank in the antibody Group. When the dispersion of antibody Group 2 is greater than the dispersion of antibody Group 1, it indicates that the globality of antibody Group 2 is better than the globality of antibody Group 1. In this case, it is only necessary to ensure that the probability of mutation of antibody Group 2 is greater than or equal to antibody Group 1. When the dispersion of antibody Group 2 is less than the dispersion of antibody Group 1, it is necessary to increase the mutation probability of antibody Group 2 to ensure that the globality of antibody Group 2 is greater than that of antibody Group 1. The mutation probability of antibody Group 2 is calculated by Equation (6).

(6) $$P_{m2}=\left\{\begin{array}{c} {a*P}_{m1}\;\;if\;P_{max2}{-P}_{min2}\geq P_{max1}-P_{min1}\\ {b*P}_{m1\;\;}if\;P_{max2}{-P}_{min2}<P_{max1}-P_{min1} \end{array}\right.$$

where $P_{m1}$ denotes the mutation probability of antibody Group 1, $P_{m2}$ denotes the mutation probability of antibody Group 2, *a*, *b* denote the multiplicity of $P_{m2}$ to $P_{m1}$, where a>1, b>a; $P_{max1}$ denotes the maximum Pareto rank of antibody Group 1, $P_{min1}$ denotes the minimum Pareto rank of antibody Group 1. $P_{max2}$ denotes the maximum Pareto rank of antibody Group 2, $P_{min2}$ denotes the minimum Pareto rank of antibody Group 2.

#### 3.1.4. Clone Inhibition

Clonal inhibition is an operation to increase the overall diversity of the population and eliminate inferior antibodies. After antibody Group 1 and antibody Group 2 undergo adaptive crossover and adaptive mutation, a new generated antibody group is introduced to further increase the diversity of the population and achieve the co-competition of the multi-antibody groups. This paper simulates the realization idea of the elite strategy [33]. Through performing non-dominated sorting and crowded-comparison operators to the multi-antibody groups, multiple outstanding individuals are selected at one time and added to the next-generation population. The remaining disadvantaged individuals are inhibited, so that the superiority of the next-generation population is ensured.

### 3.2. The Process of DAG-MOAIA

**Figure 2:**
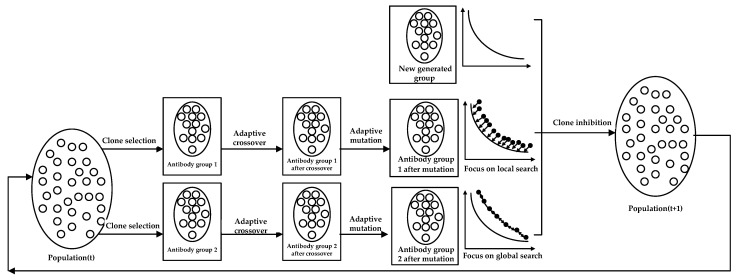
Iterative process of antibody population from generation t to generation t + 1.

Figure 2 shows the iterative process from the t-generation antibody population to the t + 1-generation. First, the antibody population is initialized. Then the antibody Group 1 and antibody Group 2 are generated by clone selection to initial antibody population. The next step is to realize independent adaptive crossover and adaptive mutation operations between different antibody groups, and obtain the offspring of antibody Group 1 and antibody Group 2 after cross-mutation. Finally, the new generated antibody group is introduced to achieve co-competition of multi-antibody groups, and through clonal inhibition, the superior antibodies are selected, and the insufficiently excellent antibodies are inhibited to form the next-generation antibody population. Algorithm 1 shows the implementation process of DAG-MOAIA.
**Algorithm 1:** DAG-MOAIA**1**Population ← Initialize the antibody population.**2****while** t < maximum number of iterations  **do****3**   Anti_group1, Anti_group2 ← Perform Clone selection to Population**4**   Anti_group1, Anti_group2 ← Perform Adaptive crossover to Anti_group1, Anti_group2**5**   Anti_group1, Anti_group2 ← Perform Adaptive mutation to Anti_group1, Anti_group2**6**   New_generated_group ← Initialize the antibody population**7**   Population ← Perform Clone inhibition to Anti_group1, Anti_group2,
New_generated_group**8**   t = t + 1**9****end while****10****Return Population**

## 4. The DAG-MOAIA for RapidIO Routing Strategy

For the specific topological structure of the RapidIO network, this section proposes a new antibody structure, tree-shaped antibody, and describes the encoding method as well as generation process of the antibody. In addition, then it describes how the operations of DAG-MOAIA are applied to the process of solving the problem of RapidIO routing strategy. The process mainly includes the generation of antibody Group 1 and antibody Group 2, the immunization of double groups, and the co-competition of multi-antibody groups.

### 4.1. Tree-Shaped Antibody

Most meta-heuristic intelligent algorithms are mainly used to optimize the continuous problem [34], while the problem of RapidIO routing strategy is to find an optimal multicast tree under multiple constraints, to minimize the cost of RapidIO packet transmission in the multicast tree. This problem belongs to the discrete problem. For the discrete problems, traditional binary coding is not able to express the antibody. This paper cites a new encoding method: two-cell coding.

The expression of the antibody is shown in Equation (7).

(7) $$Anti=\left\{cell1,cell2\right\}$$

where $cell1=\left\{x_{1},x_{2},x_{3},\ldots ,x_{n}\right\}$, $cell2=\left\{y_{1},y_{2},y_{3},\ldots ,y_{n}\right\}$, generally, $y_{n}$ is the predecessor node of $x_{n}$, when n = 1, $x_{1}$ defaults to the source node and $y_{1}$ defaults to 0.

**Figure 3:**
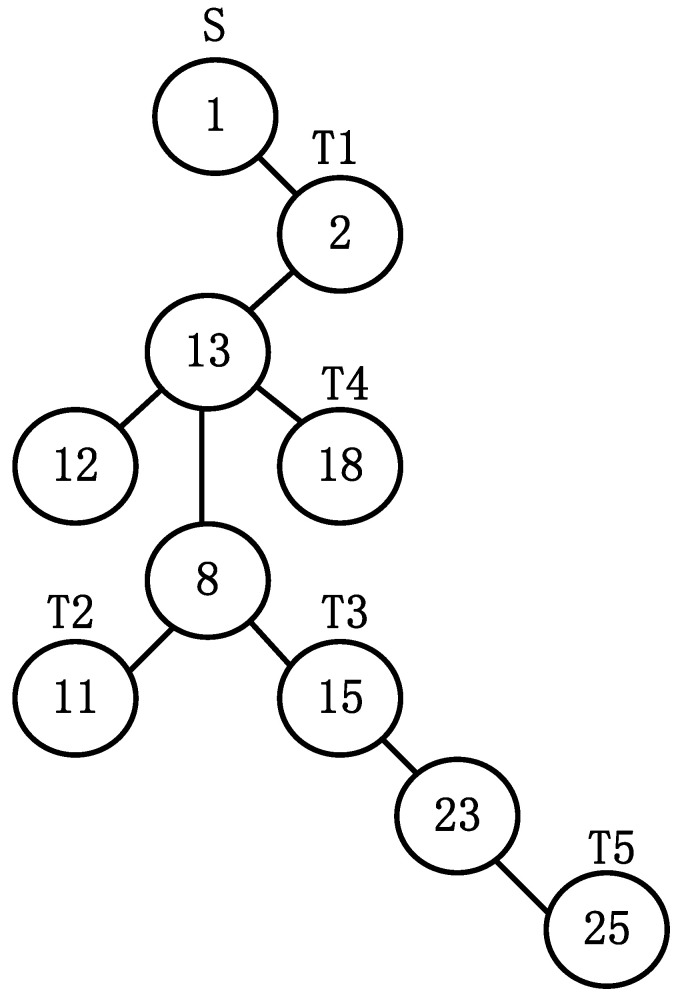
The tree-shaped antibody.

Figure 3 shows a multicast tree with source node $s=\left\{1\right\}$ and destination nodes set $T=\left\{2,\;11,\;15,\;18,\;25\right\}$.

According to the definition of two-cell coding, the coding of antibody is shown as below.

(8) $$Anti=\left\{\left(1,2,13,12,18,8,11,15,23,25\right),\right.\left(0,1,2,13,13,13,8,8,15,23\right)\}$$

The antibody is a multicast tree generated according to a given RapidIO network G, source node S, and destination nodes set E. Algorithm 2 describes the process of generating a tree-shaped antibody.
**Algorithm 2:** init_antibody(G,S,E)
**Input**: network: *G*; source node: *S*; destination nodes set: *E*
**Output**: AntiBody.**1****For** i = 1 : length(E)**2**   **If** i = 1**3**      randomly select a destination node *T* in *E***4**      Cur ← S**5**      **While** Cur≠T **do****6**         select adjacent node N to Cur, adjacent link e according to G**7**         path=path∪e; node=node∪N**8**      **End While****9**   **Else****10**     select a destination node T that has not been selected according to E**11**     Cur ← T**12**     **While** Cur ! exsit in node **do****13**        select adjacent node N to Cur, adjacent link e according to G**14**        path=path∪e; node=node∪N**15**     **End While****16**  **End If****17****End For****18**AntiBody ← {path,node}**19****Return Antibody**

### 4.2. Generation of Antibody Group 1 and Antibody Group 2

After the antibody population initialization, antibody Group 1 and antibody Group 2 are generated by applying the clone selection of DAG-MOAIA to the initial antibody population. For the multi-objective optimization problem, non-dominated sorting and crowded-comparison operators are introduced. The antibodies are ranked according to their non-dominance among each other through non-dominance sorting, and crowding distance of each antibody is obtained through crowded-comparison operators. In such a way, each antibody has two attributes: Pareto rank, and crowding distance.

**Figure 4:**
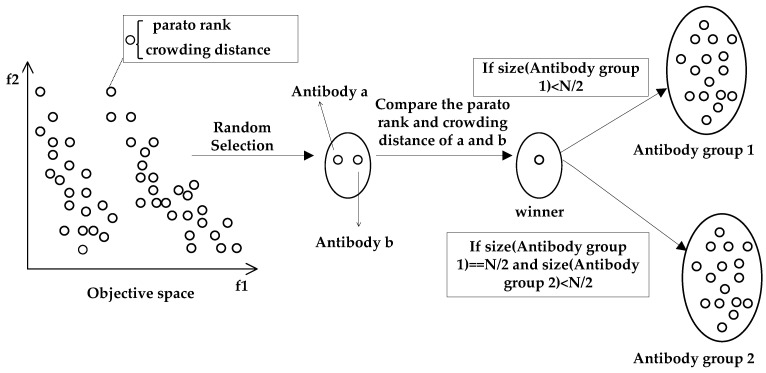
Generation process of antibody Group 1 and antibody Group 2.

Figure 4 shows the generation process of antibody Group 1 and antibody Group 2 by the clone selection. First, randomly select two antibodies at a time from the antibody population in the objective space and compare them. Since the RapidIO routing strategy problem is a minimum optimization problem, the one with the lower Pareto rank and crowding distance will be the winner and added to the next-generation population. This selection process is executed N times in total. Put the antibodies selected by first 1/2 N times into antibody Group 1, and put the antibodies selected by last 1/2 N times into antibody Group 2, ensuring the number of antibody Group 1 and antibody Group 1 are the 1/2 of initial antibody population, where N is the size of the initial antibody population. Algorithm 3 shows the process of clone selection.

### 4.3. The Immunization of Double-Antibody Groups

The immunization of double groups is mainly archived by the adaptive crossover and adaptive mutation of DAG-MOAIA, the main purpose of which is to increase the local search ability and global search ability of the antibody population. The following describes the process of implementing adaptive crossover and adaptive mutation for the RapidIO routing strategy.
**Algorithm 3:** clone_selection(Population)
**Input**: Population
**Output**: Anti_group1, Anti_group2**1**pop_rank ← Perform non-dominant sorting to population**2**pop_cdis ← Calculate the crowding distance of individuals in population.**3****For** i = 1 : 2**4**   **For** j = 1 : size(Population)/2**5**         antibody1, antibody2 ← Randomly select two antibodies from the population**6**         **If** pop_rank(antibody1) < pop_rank(antibody2)**7**            Anti_group(j) = Population(antibody1)**8**        **Else If** pop_rank(antibody1) > pop_rank(antibody2)**9**           Anti_group(j) = Population(antibody2)**10**       **Eles If** pop_ndis(antibody1) < pop_ndis(antibody2)**11**           Anti_group(j) = Population(antibody1)**12**       **Else****13**           Anti_group(j) = Population(antibody2)**14**       **End If****15**  **End for****16**  **If** i == 1**17**     Anti_group1 ← Anti_group**18**  **Else if** i == 2**19**     Anti_group2 ← Anti_group**20**  **End if****21****End for****22****Return Anti_group1, Anti_group2**

#### 4.3.1. Adaptive Crossover for the RapidIO Routing Strategy

Crossover is a process in which two parental antibodies interact with information to produce offspring. In the problem of RapidIO routing strategy, for the proposed tree-shaped antibody, the crossover operation can only be achieved by the exchange of topological structures, and then the exchange of information can be realized. However, after the partial topological structure of the two antibodies is exchanged, the connectivity of the tree-shaped antibody cannot be guaranteed. In this paper, we merge the topological structures of parental antibody 1 and parental antibody 2 to obtain the merged structure and name it ’antibody information pool’. Then the ’antibody information pool’ is regarded as a new network. A new antibody could be generated from the new network and viewed as the offspring of parental antibody 1 and parental antibody 2 after crossover.

**Figure 5:**
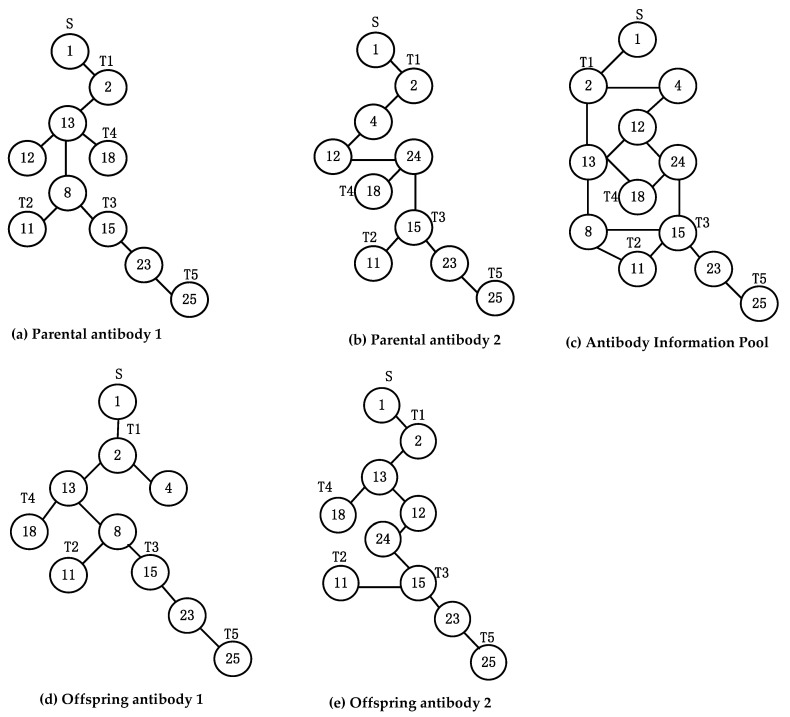
The process of crossover by parental antibodies.

In Figure 5a,b, the tree structure of parental antibody 1 and parental antibody 2 with source node $s=\left\{1\right\}$, destination nodes $T=\left\{2,\;11,\;15,\;18,\;25\right\}$ is displayed, respectively. Figure 5c represents the ’antibody information pool’ obtained by merging parental antibody 1 and parental antibody 2. Take the ’antibody information pool’ as a new network, and the offspring antibodies, offspring 1 and offspring 2, are derived from the new network as shown in Figure 5d,e. It can be found that the offspring antibodies inherit parental antibody information from different degrees, where the topology of offspring antibody 2 is inherited from parental antibody 1 and the topology of offspring antibody 1 is inherited from parental antibody 2. In such a way, this crossover operation perfectly realizes the information exchange and ensures the connectivity of the tree-shaped antibody structure. Algorithm 4 presents the process of crossover by two antibodies.
**Algorithm 4:** crossover(Antibody1, Antibody2, S, E)
**Input**: Antibody1. Antibody2, source node: *S*, destination node set: *E*
**Output**: New_antibody1, New_antibody2**1**Information_pool ← Combine Antibody1 and Antibody2**2**New_antibody1 ← inti_antibody(Information_pool, S, E)**3**New_antibody2 ← inti_antibody(Information_pool, S, E)**4****Return New_antibody1, New_antibody2**

To increase the local search capability to find better solutions, after realizing the crossover operation between each antibody, the crossover probability will be dynamically adjusted to realize the adaptive crossover operation according to the Pareto rank of the antibody. Algorithm 5 shows the adaptive crossover process.
**Algorithm 5:** adaptive_crossover(n, Anti_group, $P_{cmax}$, $P_{cmin}$)
**Input**: group type: *n*, Anti_group, source node: *S*, destination node set: *E*
            max probability of crossover:$P_{cmax}$, min probability of crossover:$P_{cmax}$,
**Output**: Off_group**1**pop_rank ← Perform non-dominant sorting to Anti_group**2**times = size(Anti_group)/2**3****While** i < times  **do****4**   antibody a, antibody b ← Randomly select two different antibodies from Anti_group**5**   //ensure that each antibody is selected only once**6**   remove antibody a and antibody b from Anti_group**7**   **If** n==1    //n==1 represents antibody group 1**8**       $P_{c}$← Calculate the crossover probability according to Equation (4) and pop_rank**9**   **Else if** n==2    //n==2 represents antibody Group 2**10**      $P_{c}$← Calculate the crossover probability according to Equation (5) and pop_rank**11**  **End if****12**  *r*← Randomly generated probability**13**  **If** *r*<=
$P_{c}$**14**     antibody_a,antibody_b=crossover(antibody_a, antibody_b, S, E)**15**  **End if****16**  Off_group(2*i-1)=antibody_a**17**  Off_group(2*i)=antibody_b**18**   i = i + 1**19****End while****20****Return Off_group**

#### 4.3.2. Adaptive Mutation for the RapidIO Routing Strategy

Mutation is an operation by which an individual changes its initial structural state. Additionally, in the RapidIO routing strategy problem for the tree-shaped antibody, changing the structure may result in the topology being unconnected or even invalid. To avoid this, the mutation process mimics the crossover process of antibodies. First, a new antibody is initialized, whose aim is to form an ’antibody information pool’ with the antibody to be mutated. Then, a new antibody is derived based on the ’antibody information pool’ and viewed as the mutated antibody. In such a way, the mutation operation based on tree-shaped antibodies is archived. Algorithm 6 is the pseudo-code of the mutation operation.
**Algorithm 6:** mutation(Antibody, G, S, E)
**Input**: Antibody, network: *G*, source node: *S*, destination nodes set: *E*
**Output**: New_antibody**1**Temp_antibody ← init_antibody(G, S, E)**2**Information_pool ← Combine New_antibody and Antibody**3**New_antibody ← inti_antibody(Information_pool, S, E)**4****Return New_antibody**

For improving the global search capability to increase the solution space, after realizing the mutation operation of each antibody, the mutation probability will be dynamically adjusted to realize the adaptive mutation operation according to the relative diversity between the antibody Group 1 and antibody Group 2. Algorithm 7 shows the adaptive mutation process.
**Algorithm 7:** adaptive_mutation(n, Anti_group, $P_{max}$, $P_{min}$, $P_{m1}$, G, S, E)
**Input**: group type: *n*, Anti_group, network: *G*, source node: *S*, destination nodes set: *E*
           max probability of mutation:$P_{cmax}$, min probability of mutation:$P_{cmax}$
           mutation of Antigroup1: $P_{m1}$
**Output**: Off_group**1**times = size(Anti_group)**2****While** i < times**3**   antibody a ← Randomly select an antibody from Anti_group**4**   //ensure that each antibody is selected only once**5**   remove antibody a from Anti_group**6**   **If** n==1    //n==1 represents antibody group 1**7**      $P_{m}$←$P_{m1}$**8**   **Else if** n==2    //n==2 represents antibody group 1**9**      $P_{m}$← Calculate the crossover probability according to Equation (6)**10**  **End if****11**  *r*← Randomly generated probability**12**  **If** *r*<=
$P_{m}$**13**     antibody_a = mutation(antibody_a, G, S, E)**14**  **End if****15**  Off_group(i)=antibody_a**16**   i = i + 1**17****End While****18****Return Off_group**

### 4.4. The Co-Competition of Multi-Antibody Groups

The co-competition of multi-antibody groups is achieved by clonal inhibition of DAG-MOAIA, the purpose of which is to further maintain the diversity of the population. A new generated antibody group is introduced to form the merged antibody groups with antibody Group 1 and antibody Group 2. Then the merged antibody groups will be sorted from small to large according to the Pareto rank of each antibody. For the antibodies with equal Pareto rank, they will be sorted again according to the crowding distance. Finally, the top N excellent antibodies will be selected to join the next population, and the rest will be inhibited.

**Figure 6:**
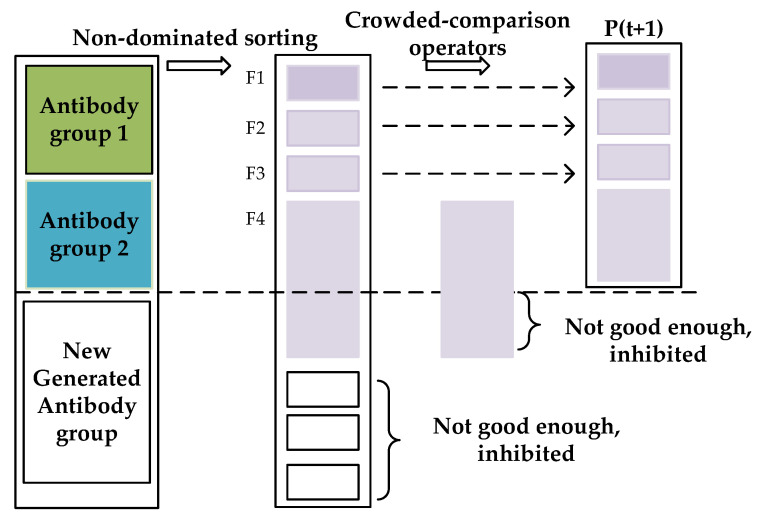
The process of clone inhibition to multiple groups.

Figure 6 shows the specific implementation process of generating the next antibody population through clonal inhibition. Among the antibody Group 1, antibody Group 2 and new generated antibody group, the front F1, F2, and F3 of Pareto optimal solutions are selected to the next population through non-dominated sorting. For F4, if it is added, then the overall number will exceed the size of the initial antibody population. Therefore, the crowded-comparison operator is used to select the more superior individuals and inhibit the inferior ones, so that the number of the next population is finally maintained to be equal to the initial antibody population size. Algorithm 8 presents the pseudo code of clone inhibition process.
**Algorithm 8:** clone_inhibition(Anti_group1,Anti_group2,New_generated_group, N)
**Input**: Anti_group1, Anti_group2, New_generated_group, population size: *N*
**Output**: Population**1**pop_rank ← Perform non-dominant sorting to population**2**pop_cdis ← Calculate the crowding distance of individuals in population.**3**New_population ← Combine Anti_group1, Anti_group2, $New\_generated_{g}roup$**4**Update New_population by sorting New_population in ascending order according to pop_rank**5**Update New_population by sorting New_population in ascending order according to pop_cids**6**Population ← New_population(1:N)**7****Return Population**

## 5. Simulation Experiment

This experiment uses Matlab programming to implement the DAG-MOAIA proposed in this paper. The hardware environment is Intel(R) Core(TM) i5-7200U CPU @ 2.50 GHz, 2.70 GHz.8.9 GBRAM, 512 G hard disk, and the development tool used is MATLAB R2019b.

Meanwhile, to examine the performance of DAG-MOAIA, this experiment randomly generates 10 network topologies: RDNet-1 RDNet10. Each network topology contains nodes between 20 and 60. The specifications of the random network topology are shown in Table 1.

To further evaluate the performance of DAG-MOAIA, the currently popular multi-objective optimization algorithms including NSGA-II [35], MOPSO [36], MOMRDE [37] are compared with DAG-MOAIA. Then, to better reflect the improvement of DAG-MOAIA, this paper compares MOAIA with DAG-MOAIA in the experiment. In contrast to DAG-MOAIA, MOAIA adopts the strategy of single antibody group immunization. During the immunization process, the crossover and mutation of probability are all fixed values, rather than adaptively adjusting during the immunization process similar to DAG-MOAIA. Table 2 describes the public experimental parameter settings, and Table 3 shows the parameter settings of all algorithms.

Table 3 introduces the parameter settings of different algorithms in the experiment. In MOMRDE, $c_{r}$ represents the crossover probability, *K* represents the scale factor, and $I_{c}$ represents the isocapacity; In NSGA-II, $p_{c}$ represents the crossover probability, and $p_{m}$ represents the variation probability; In the MOPSO, *w* represents inertia coefficient, $c_{1}$ represents individual cognitive coefficient, $c_{2}$ represents social cognitive coefficient; In MOAIA, $p_{c}$ represents crossover probability, and $p_{m}$ represents mutation probability.

### 5.1. Evaluation Indicators for MOP

To evaluate the comprehensive performance of each aspect of the algorithm, define $PF_{k}$ to denote the Pareto front corresponding to Pareto sets obtained by each algorithm, $PF_{r}$ to denote the true Pareto front corresponding to Pareto sets from all algorithms. Regard $PF_{r}$ as reference for $PF_{k}$. The following relevant performance metrics are used in the experiments of this paper, which are widely recognized in the literature [38,39] as well.

1. HV(Hyper Volume)
  HV is used to measure the coverage of Pareto sets obtained by the algorithm in the objective space and is defined as follows.
  (9) $$HV=\sigma \left(\cup_{i=1}^{|{PF}_{r}|}v_{i}\right)$$
  where σ represents the Lebesgue [40] measure and is used to measure the volume, $PF_{r}$ denotes the total number of Pareto sets, $v_{i}$ denotes the hyperspace constituted by the ith solution in the reference point to the solution in Pareto sets. A larger HV value indicates that the Pareto sets obtained by the algorithm cover a wider area in the objective space, are more evenly distributed and are closer to the true Pareto front.
2. IGD (Inverted Generational distance)
  (10) $$IGD=\frac{\sum_{v\in {PF}_{r}}d\left(v,{PF}_{k}\right)}{\left|{PF}_{r}\right|}$$
  where $d\left(v,{PF}_{k}\right)$ denotes the minimum Euclidean distance between an individual *v* in ${PF}_{r}$ and its nearest ${PF}_{k}$, $\left|{PF}_{r}\right|$ denotes the total number of true Pareto front. IGD measures both the convergence and diversity of the algorithm. A smaller IGD can better reflect the overall optimization performance of the algorithm.

### 5.2. Experimental Results and Analysis

This section will verify the performance of DAG-MOAIA from three aspects: the comparison of Pareto front, the optimization of QoS constraints, and the comparison of HV and IGD.

#### 5.2.1. The Comparison of Pareto Front

**Figure 7:**
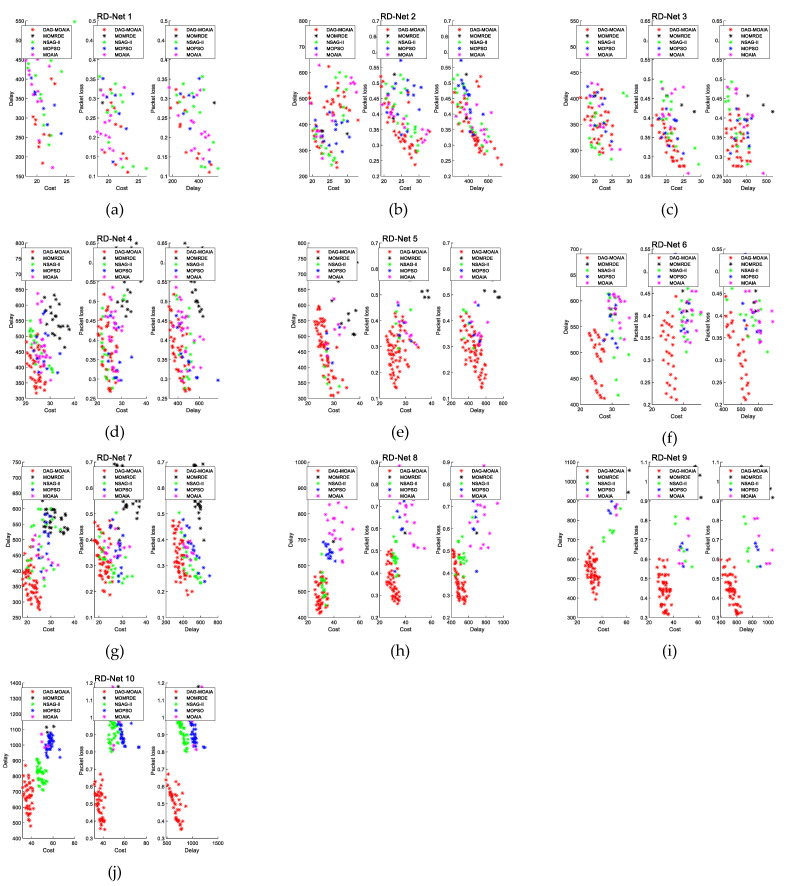
The comparison of bi-objective in the Pareto front. (**a**) The comparison of bi-objective in the RD_Net 1. (**b**) The comparison of bi-objective in the RD_Net 2. (**c**) The comparison of bi-objective in the RD_Net 3. (**d**) The comparison of bi-objective in the RD_Net 4. (**e**) The comparison of bi-objective in the RD_Net 5. (**f**) The comparison of bi-objective in the RD_Net 6. (**g**) The comparison of bi-objective in the RD_Net 7. (**h**) The comparison of bi-objective in the RD_Net 8. (**i**) The comparison of bi-objective in the RD_Net 9. (**j**) The comparison of bi-objective in the RD_Net 10.

**Figure 8:**
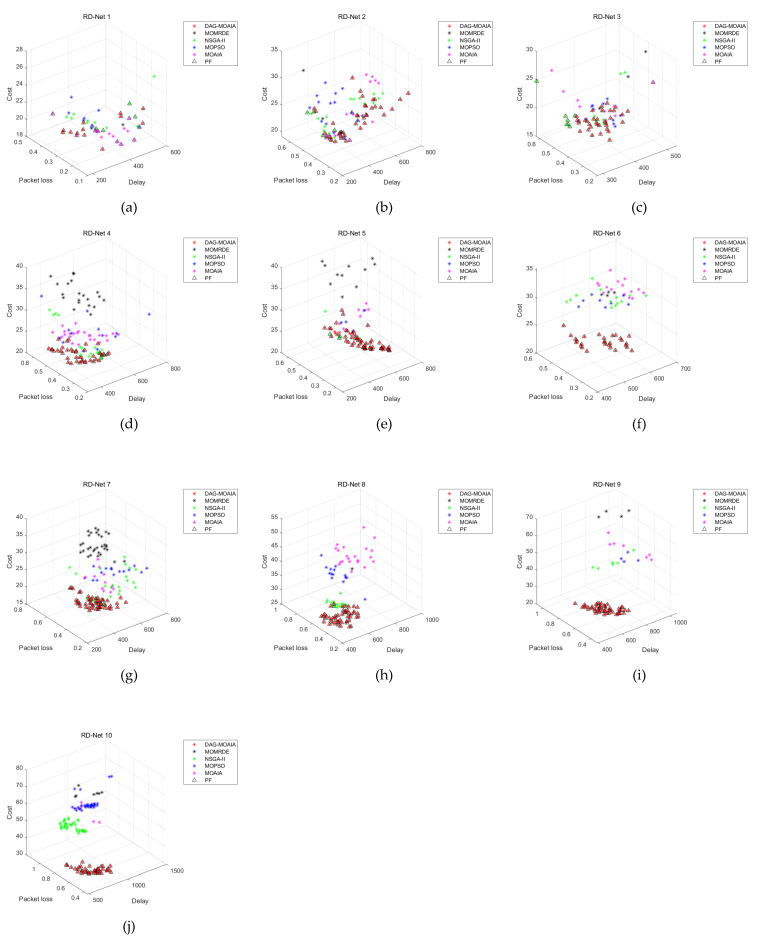
The comparison of Pareto front in three dimensions. (**a**) The comparison of the Pareto front in the RD_Net 1. (**b**) The comparison of the Pareto front in the RD_Net 2. (**c**) The comparison of the Pareto front in the RD_Net 3. (**d**) The comparison of the Pareto front in the RD_Net 4. (**e**) The comparison of the Pareto front in the RD_Net 5. (**f**) The comparison of the Pareto front in the RD_Net 6. (**g**) The comparison of the Pareto front in the RD_Net 7. (**h**) The comparison of the Pareto front in the RD_Net 8. (**i**) The comparison of the Pareto front in the RD_Net 9. (**j**) The comparison of the Pareto front in the RD_Net 10.

Figure 7 demonstrates the bi-objective comparison of the Pareto front corresponding to Pareto sets obtained by different algorithms in different networks. Figure 8 directly shows the comparison of the Pareto front corresponding to Pareto sets in the three-dimensional space. Take the Pareto sets obtained by all algorithms as the overall solution set, then find Pareto sets based on overall solution set, and regard it as the true Pareto front, denoted as △.

It can be found from Figure 7a–d that when the number of nodes in the network is not much and there are few destination nodes, DAG-MOAIA does not show much advantage over other algorithms. The optimization objectives of the solution sets of each algorithm are mutually dominated. However, analyzing together with Figure 8a,d, it can be found that the Pareto front obtained by DAG-MOAIA overlap highly with △, which indicates that the majority of solutions in Pareto sets is obtained by DAG-MOAIA dominating those obtained by the other algorithms, It can be concluded that DAG-MOAIA may not differ much from other algorithms in bi-objective optimization when the network is simple and the number of target nodes is small, but in combination with simultaneous analysis of treble objectives, DAG-MOAIA still outperforms other algorithms.

As the number of network nodes and the destination nodes increase, the network becomes complex. As can be seen from Figure 7e–j and Figure 8e–j, it is obvious that DAG-MOAIA is superior to other algorithms, whether from the comparison of the Pareto front or from the comparison of bi-objective of the Pareto front. In comparison to the bi-objective of the Pareto front, the Pareto sets obtained by DAG-MOAIA directly dominate the Pareto sets obtained by the other algorithms, illustrating that DAG-MOAIA has a more integrated performance in the optimization process compared to the other algorithms. In the direct comparison of Pareto front, the quality of solution in the Pareto set obtained by DAG-MOAIA is better than the quality of those obtained by the other algorithms, indicating that DAG-MOAIA could jump out of the local optimum to find better solutions. By comparing the number of solutions, the number of solutions obtained by DAG-MOAIA is also higher than the number of solutions obtained by the other algorithms, showing that DAG-MOAIA has better diversity.

#### 5.2.2. The Optimization of QoS Constraints

This section evaluates the performance of different algorithms by presenting the optimization effect on QoS constraints such as packet loss rate, delay and transmission cost.

**Figure 9:**
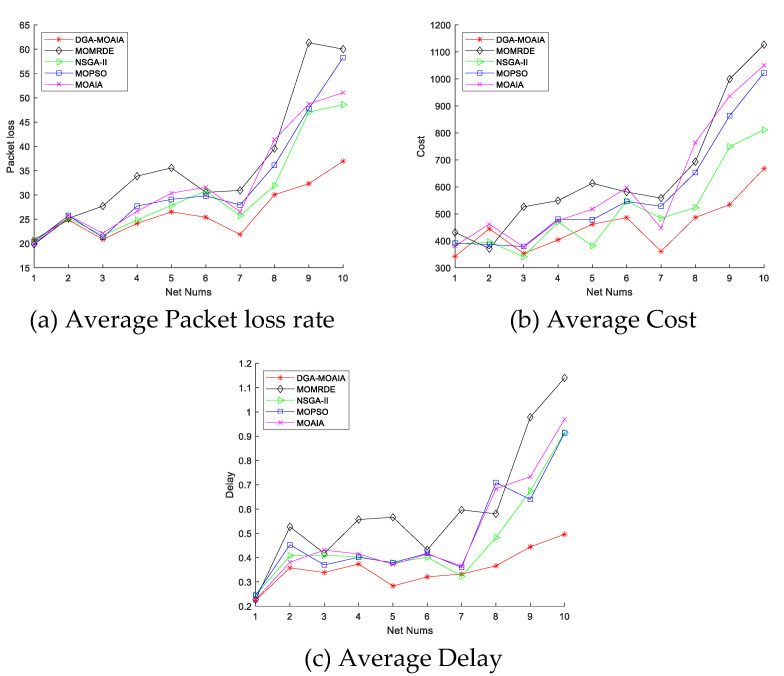
The comparison of average transmission delay, average transmission cost, and average packet loss rate.

Figure 9 shows the comparison of the average packet loss rate, transmission cost, and delay of different algorithms in the 10 networks. It can be found that as the number of network nodes as well as the number of destination nodes increases, and the network becomes increasingly complex, which affects the performance of the algorithm to some extent. In addition, as the network size increases, the average transmission delay, cost, and packet loss rate of different algorithms also increases gradually. However, overall, DAG-MOAIA still outperforms other algorithms. The packet loss rate, delay and transmission cost obtained by DAG-MOAIA during data transmission in the RapidIO network are lower than those obtained by other algorithms, which suggests that DAG-MOAIA can find a better transmission path, making the data transmission with least cost. This also indirectly indicates that DAG-MOAIA obtains solutions with better quality and has better performance.

#### 5.2.3. The Comparison of HV and IGD

In this section, the average values of HV and IGD obtained by different algorithms are compared to test the quality of the solution obtained by the algorithm, as well as the diversity, convergence and comprehensive performance of the algorithm.

The average values of HV and IGD obtained by different algorithms in different networks are shown in Table 4 and Table 5. In Table 4, it can be found that DAG-MOAIA has a higher average HV value than other algorithms in different networks in almost all cases, indicating that the solutions in Pareto sets obtained by DAG-MOAIA are closer to the true Pareto front than other algorithms, also explaining that the quality of its solution is better than those obtained by other algorithms. In Table 5, the average values of IGD obtained by DAG-MOAIA are smaller than those obtained by other algorithms, which provides an explanation that DAG-MOAIA not only has better convergence and diversity, but also better comprehensive performance.

## 6. Discussion

In this paper, we propose DAG-MOAIA for the RapidIO routing strategy problem during packet transmission in the RapidIO network, and put forward adaptive crossover operator and adaptive mutation operator for the first time in a multi-objective problem to improve the local search ability and global search ability. In addition, we further increase the diversity of population by achieving co-competition of multi-antibody groups. We have evaluated the performance of DAG-MOAIA in different aspects.

First, by comparing the Pareto front corresponding to Pareto sets obtained by different algorithms, it is found that the solutions obtained by DAG-MOAIA are not only better in quality, but also more in quantity than those obtained by other algorithms, which explains that DAG-MOAIA has better search ability to find the optimal solution and better diversity of algorithms; then, by contrast with the optimization effects of different QoS constraint parameters, the DAG-MOAIA could find a better transmission path, which accounts for the lower transmission cost of data packets in the RapidIO network, and the higher quality of solutions obtained by DAG-MOAIA. Finally, the evaluation indicators of MOP, such as IGD and HV, are introduced to analyze the diversity, convergence and comprehensive performance of the different algorithms. From the data in Table 4 and Table 5, it can be concluded that the solutions obtained by DAG-MOAIA are closer to the true Pareto front, and the diversity, convergence and comprehensive performance of DAG-MOAIA are better than other algorithms.

In summary, the quality of the solutions obtained by DAG-MOAIA is higher compared to other algorithms. The DAG-MOAIA can select better transmission paths and obtain better performance. In the further work, we will continue to develop the algorithm for solving the problem of RapidIO routing strategy. In addition, we will apply DAG-MOAIA to other areas such as Telematics, IoT, Edge Computing, etc. 

## Figures and Tables

**Table 1:** Random network topology specifications.

Network	Number of Nodes	Number of Target Nodes
RDNet-1	20	5
RDNet-2	20	7
RDNet-3	30	6
RDNet-4	30	7
RDNet-5	40	6
RDNet-6	40	8
RDNet-7	50	8
RDNet-8	50	10
RDNet-9	60	12
RDNet-10	60	14

**Table 2:** Public experimental parameter settings.

Relevant Parameters	Parameter Settings
population size	100
iteration times	200
packet loss rate	(0, 1)
transmission cost	depends on the Euclidean distance between the nodes
link delay	10 ms–100 ms

**Table 3:** Parameter settings of all algorithms.

Algorithm	Relevant Parameter	Parameter Settings
DAG-MOAIA	$p_{cmax1}$	0.8
	$p_{cmax1}$	0.2
	$p_{cmin1}$	0.5
	$p_{cmax2}$	0.2
	$p_{m1}$	0.5
	*a*	1
	*b*	2
MOMRDE	$c_{r}$	0.8
	*K*	0.3
	$I_{c}$	1.0 × 10${}^{-4}$
NSGA-II	$p_{c}$	0.7
	$p_{m}$	0.3
MOPSO	*w*	0.4
	$c_{1}$	2
	$c_{2}$	2
MOAIA	$p_{c}$	0.8
	$p_{m}$	0.2

**Table 4:** Mean values of HV (best results are shown in bold).

Net	DAG-MOAIA	MOMRDE	NSGA-II	MOPSO	MOAIA
RD-Net1	**0.4621**	0.3383	0.4421	0.4238	0.4397
RD-Net2	**0.4226**	0.3274	0.3984	0.3692	0.3925
RD-Net3	**0.3779**	0.2398	0.3464	0.3110	0.3316
RD-Net4	**0.3541**	0.2394	0.3253	0.2994	0.3053
RD-Net5	**0.3982**	0.1916	0.3208	0.2674	0.2939
RD-Net6	**0.3232**	0.1370	0.2368	0.2142	0.1994
RD-Net7	**0.3841**	0.1597	0.3426	0.2869	0.2935
RD-Net8	**0.3626**	0.1245	0.2599	0.1901	0.1754
RD-Net9	**0.3841**	0.0491	0.1853	0.1093	0.1057
RD-Net10	**0.3605**	0.0701	0.1794	0.1503	0.1586

**Table 5:** Mean values of IDG (best results are shown in bold).

Net	DAG-MOAIA	MOMRDE	NSGA-II	MOPSO	MOAIA
RD-Net1	**2.0392**	40.1387	4.6520	9.9142	9.7219
RD-Net2	**3.1534**	47.1729	14.5590	14.9630	11.1055
RD-Net3	**1.1551**	45.5954	9.9934	13.2271	10.6940
RD-Net4	**1.8198**	35.3617	9.5404	14.5186	9.9136
RD-Net5	**0.3844**	94.2895	24.5864	36.5975	35.3348
RD-Net6	**0.0807**	171.6898	30.1431	52.1168	72.0818
RD-Net7	**0.8119**	129.9506	9.9466	23.4085	18.5274
RD-Net8	**0.0240**	157.3212	33.2815	157.7992	148.0127
RD-Net9	**0.0000**	407.5112	107.7797	286.7245	270.5150
RD-Net10	**0.0000**	350.5060	57.3034	130.9448	136.7497

## Data Availability

No applicable.
